# Prognostic Factors in Primary Biliary Cholangitis: A Retrospective Study of Joint Slovak and Croatian Cohort of 249 Patients

**DOI:** 10.3390/jpm11060495

**Published:** 2021-06-01

**Authors:** Jakub Gazda, Sylvia Drazilova, Martin Janicko, Ivica Grgurevic, Tajana Filipec Kanizaj, Tomas Koller, Beatrica Bodorovska, Tonci Bozin, Maja Mijic, Zrinka Rob, Ivana Mikolasevic, Anita Madir, Branislav Kucinsky, Peter Jarcuska

**Affiliations:** 1 2nd Department of Internal Medicine, PJ Safarik University in Kosice and L. Pasteur University Hospital, Trieda SNP 1, 040 11 Kosice, Slovakia; jakub.gazda@upjs.sk (J.G.); martin.janicko@gmail.com (M.J.); brano1989@gmail.com (B.K.); peter.jarcuska@upjs.sk (P.J.); 2 Internal Medicine Department, Hospital Poprad, a.s., 058 01 Poprad, Slovakia; 3 Department of Gastroenterology, Hepatology and Clinical Nutrition, University Hospital Dubrava, Avenija Gojka Suska 6, 10000 Zagreb, Croatia; ivica.grgurevic@zg.htnet.hr (I.G.); tbozin@gmail.com (T.B.); zrinka_rob@yahoo.com (Z.R.); 4 Department of Gastroenterology, Hepatology and Clinical Nutrition, School of Medicine, University of Zagreb, Salata ul. 2, 10000 Zagreb, Croatia; tajana.filipec@gmail.com (T.F.K.); anita.madir@gmail.com (A.M.); 5 Department of Gastroenterology, University Hospital Merkur, Zajceva ul. 19, 10000 Zagreb, Croatia; mijic.maja@gmail.com (M.M.); ivana.mikolasevic@gmail.com (I.M.); 6 5th Department of Internal Medicine, Subdivision of Gastroenterology and Hepatology, Comenius University Faculty of Medicine, University Hospital Bratislava, Ruzinovska 6, 826 06 Bratislava, Slovakia; tomas.koller@fmed.uniba.sk; 7 Clinic of Gastroenterological Internal Medicine, Comenius University in Bratislava and University Hospital Martin, Kollarova 2, 036 59 Martin, Slovakia; beatrica.bodorovska@gmail.com; 8 Department of Gastroenterology, School of Medicine, University of Rijeka, Brace Branchetta 20/1, 51000 Rijeka, Croatia

**Keywords:** primary biliary cholangitis, ursodeoxycholic acid, prognostic factors, treatment response, liver decompensation

## Abstract

Objective: To identify pretreatment laboratory parameters associated with treatment response and to describe the relationship between treatment response and liver decompensation in patients with primary biliary cholangitis treated with ursodeoxycholic acid. Methods: We defined treatment response as both ALP ≤ 1.67 × ULN and total bilirubin ≤ 2 × ULN. Multiple logistic regression analyses were performed to adjust for confounding effects of sociodemographic variables. Results: Pretreatment total bilirubin ((TB); OR = 0.3388, 95%CI = 0.1671–0.6077), ALT (OR = 0.5306, 95%CI = 0.3830–0.7080), AST (OR = 0.4065, 95%CI = 0.2690–0.5834), ALP (OR = 0.3440, 95%CI = 0.2356–0.4723), total cholesterol ((TC); OR = 0.7730, 95%CI = 0.6242–0.9271), APRI (OR = 0.3375, 95%CI = 0.1833–0.5774), as well as pretreatment albumin (OR = 1.1612, 95%CI = 1.0706–1.2688) and ALT/ALP (OR = 2.4596, 95%CI = 1.2095–5.5472) were associated with treatment response after six months of treatment. Pretreatment TB (OR = 0.2777, 95%CI = 0.1288–0.5228), ALT (OR = 0.5968, 95%CI = 0.4354–0.7963), AST (OR = 0.4161, 95%CI = 0.2736–0.6076), ALP (OR = 0.4676, 95%CI = 0.3487–0.6048), APRI (OR = 0.2838, 95%CI = 0.1433–0.5141), as well as pretreatment albumin (OR = 1.2359, 95%CI = 1.1257–1.3714) and platelet count (OR = 1.0056, 95%CI = 1.0011–1.0103) were associated with treatment response after 12 months of treatment. Treatment response after 6 months of UDCA therapy is significantly associated with treatment response after 12 months of UDCA therapy (OR = 25.2976, 95% CI = 10.5881–68.4917). Treatment responses after 6 and 12 months of UDCA therapy decrease the risk of an episode of liver decompensation in PBC patients (OR = 12.1156, 95%CI = 3.7192–54.4826 and OR = 21.6000, 95%CI = 6.6319–97.3840, respectively). Conclusions: There are several pretreatment laboratory parameters associated with treatment response in patients with primary biliary cholangitis. Treatment response after six months is significantly associated with treatment response after 12 months of ursodeoxycholic acid (UDCA) therapy. Treatment responses after 6 and 12 months of UDCA decrease the risk of an episode of liver decompensation.

## 1. Introduction

Primary biliary cholangitis (PBC) is a chronic autoimmune cholestatic liver disease leading to the destruction of biliary epithelial cells, subsequent cholestasis, and progressive biliary fibrosis. PBC may culminate in liver cirrhosis and subsequent development of hepatocellular carcinoma. Both liver decompensation and hepatocellular carcinoma may result in liver-related death [1].

PBC prevalence varies depending on region and ranges from 1.9 to 40.2 per 100,000 inhabitants, while the incidence ranges from 0.3 to 5.8 per 100,000 inhabitants [2]. In Slovakia, the 2019 point-prevalence was 14.9 cases per 100,000 inhabitants, while the annual incidence during recent years ranged from 0.7 to 1.5 per 100,000 inhabitants [3]. In Croatia, the point prevalence ranged from 11.5 to 12.5 per 100,000 inhabitants (depending on region), and the average incidence was 0.79 to 0.89 per 100,000 inhabitants [4]. Interestingly, with earlier diagnosis and decreasing mortality due to more effective treatment, PBC prevalence increased during the last couple of years [5]. PBC is female-predominant and male patients account only for 3%–24% of all patients [2]. 

Antimitochondrial antibodies M2 (AMA M2) are present in 95% of patients, and antinuclear antibodies (anti-gp210/anti-sp100) are present frequently as well [6]. An increased level of alkaline phosphatase (ALP) is another typical laboratory finding. For PBC diagnosis, patients have to meet at least two out of the three following criteria: (1) increased ALP, (2a) the presence of AMA at a titer >1:40 or (2b) the presence of anti-sp100/antigp210, and (3) histological signs [1]. 

Fatigue and pruritus are the most common symptoms, however, approximately one-fourth of patients is completely asymptomatic. PBC is frequently associated with other autoimmune liver disorders (autoimmune hepatitis and primary sclerosing cholangitis) and with extrahepatic autoimmune diseases (autoimmune thyroiditis, coeliac disease, or Sjogren syndrome) [3]. 

Ursodeoxycholic acid (UDCA) is the first-line pharmacotherapy in all patients with PBC (13–15 mg/kg/day) [1]. The first-line treatment is well tolerated, and approximately 70% of patients respond to it. Obeticholic acid (OCA) or fibrates can be offered to non-responders, however, fibrates are currently off-label [7,8]. This study aims to identify baseline factors associated with the treatment response to UDCA after both 6 and 12 months of therapy using a cohort of Slovak and Croatian patients with PBC.

## 2. Materials and Methods

### 2.1. Study Design

We conducted a multicenter retrospective study of PBC patients to describe pretreatment differences between responders and non-responders to UDCA. Furthermore, we aimed to identify factors related to treatment response and evaluate the relationship between treatment response and liver decompensation.

Patients included in the study were treated for PBC in 11 different hepatology centers in Slovakia and Croatia during the period from 30 June 1999, through 30 June 2019. The exclusion criteria were: (a) insufficient data for the verification of PBC diagnosis, (b) concomitant liver disease, (c) liver transplantation after less than 12 months of UDCA treatment, (d) immunosuppressive treatment or OCA, and (e) follow up less than six months.

Local investigators completed case report forms (CRF) with the on-call assistance from the study coordinators and collected detailed pretreatment demographic and clinical information as follows: age, sex, biochemical, hematological, and immunological profile, histological assessment where available, and the initial UDCA dosage. Additionally, CRF included data necessary for evaluating the treatment response after 6 and after 12 months of UDCA therapy, APRI after 12 months of UDCA therapy, duration of the follow-up, history of liver decompensation, liver transplantation, and liver-related mortality. To account for interlaboratory variability, total bilirubin (TB), aspartate aminotransferase (AST), alanine aminotransferase (ALT), and alkalic phosphatase (ALP) were all transformed into multiple of their respective upper limit of normal (ULN).

All completed CRFs were centrally evaluated for the confirmation of PBC diagnosis. The European Association for the Study of the Liver (EASL) recommends that two out of three following criteria need to be met: (1) elevated ALP, (2a) presence of AMA at a titer >1:40 or (2b) anti-sp100/antigp210, and (3) histological signs after liver biopsy.

We defined the treatment response as ALP ≤ 1.67 × ULN and total bilirubin ≤ 2 × ULN and evaluated patients for its achievement after 6 and 12 months of UDCA therapy. Furthermore, we specified liver decompensation as history of either ascites, variceal bleeding, and hepatic encephalopathy.

The study protocol is in accordance with the 1964 Helsinki declaration, its later amendments, and the principles of good clinical practice. The study protocol was approved by the Ethical committee of Poprad Hospital, a.s. on 5 May 2019. The committee waived the need for the specific patients’ informed consent due to the retrospective nature of the data collection.

### 2.2. Statistical Analysis

We checked the normality of the distribution of continuous variables using Shapiro-Wilk tests. Continuous variables are described by medians and interquartile ranges (IQR) because most of them were not normally distributed. Categorical variables are described as absolute counts and percentages. We excluded variables with a large proportion of missing data (>16%) from further statistical analyses. A non-parametric “*k*-nearest neighbors” (*k*NN) algorithm was used to perform data imputation because the rest of the missing values were “missing at random”. *k*NN is a machine learning method that is useful for matching a point with its *k* closest neighbors in a multi-dimensional space. The *k*NN algorithm can be used for imputing missing data by finding the *k* closest neighbors to the observation with missing data and then imputing them based on the means of the non-missing values in the neighbors. We set the value of the *k* to be 20. Mann-Whitney U tests were used to compare differences in continuous variables. χ2 and McNemar‘s tests were used to compare differences in categorical variables (latter in the case of paired data). We performed multiple logistic regression analyses to search for variables associated with the treatment response after age and sex adjustment and to describe the relationship between treatment response achievement and liver decompensation. We checked influential observations for their validity. The results of regression analyses are presented as odds ratios (OR) with 95% confidence intervals (95%CI) and *p*-values. All tests were two-sided and performed at a 0.05 significance level. Data were analyzed using RStudio (version 1.2.1335).

## 3. Results

**Figure 1:**
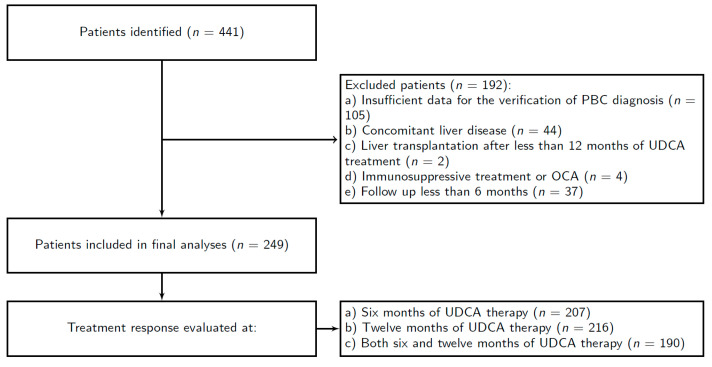
Flowchart of patients’ inclusion.

Initially, we excluded 192 patients due to meeting exclusion criteria and subsequently included 249 patients in the final statistical analysis (Figure 1). Furthermore, we excluded the following variables from the analysis due to a large proportion of missing data: C-reactive protein (CRP), direct bilirubin, ferritin, gamma-glutamyltransferase (GGT), high-density lipoprotein cholesterol (HDL-C), immunoglobulin M (IgM), low-density lipoprotein cholesterol (LDL-C), prothrombin time (PT), the ratio of absolute counts of neutrophils and lymphocytes (Neu/Ly), and triglycerides (TG).

We analyzed data of 234 female and 15 male patients (94% and 6%, respectively) with the median pretreatment age of 56.00 years (IQR 13). In total, 15 female patients (6.4%) were 40 years old or younger. A total of 79 patients (31.7%) had significant pretreatment liver fibrosis as defined by APRI > 0.7 [9]. The median UDCA dosage was 1000 mg per day (IQR 500). We evaluated 207 patients (83.1%) after six months and 216 patients (86.8%) after 12 months of therapy for the treatment response to UDCA. In total, 190 patients (76.3%) were evaluated for the treatment response to UDCA after both 6 and 12 months of therapy. The median duration of the follow-up was five years (IQR 6). A total of 20 patients (8%) experienced an episode of liver decompensation during the follow-up, five of these patients (25%) underwent liver transplantation, and 10 patients died (50%) (Table 1).

We identified several significant pretreatment differences between responders and non-responders after six months of UDCA therapy (Table 2). Responders had significantly lower pretreatment levels of total bilirubin (*p* = 0.03), ALT (*p* = 0.0001), AST (*p* = 0.00002), ALP (*p* < 0.00001), TC (*p* = 0.03), and APRI (*p* = 0.00003) and they also had significantly higher pretreatment levels of albumin (*p* = 0.005) and ALT/ALP (*p* = 0.00004).

Furthermore, we did multiple logistic regression analyses to identify pretreatment factors associated with the treatment response after six months of UDCA therapy (Table 3). Lower pretreatment total bilirubin (OR 0.3388, 95%CI 0.1671–0.6077), ALT (OR 0.5306, 95%CI 0.3830–0.7080), AST (OR 0.4065, 95%CI 0.2690–0.5834), ALP (OR 0.3440, 95%CI 0.2356–0.4723), TC (OR 0.7730, 95%CI 0.6242–0.9271), APRI (OR 0.3375, 95%CI 0.1833–0.5774), as well as higher pretreatment albumin (OR 1.1612, 95%CI 1.0706–1.2688) and ALT/ALP (OR 2.4596, 95%CI 1.2095–5.5472) were after age and sex adjustment associated with higher probability of treatment response after 6 months of UDCA therapy.

We also identified several significant pretreatment differences between responders and non-responders after 12 months of UDCA therapy (Table 4). Responders had significantly lower pretreatment levels of ALT (*p* = 0.001), AST (*p* = 0.0001), ALP (*p* < 0.00001), and APRI (*p* = 0.0001), and they also had significantly higher pretreatment levels of albumin (*p* = 0.001) and ALT/ALP (*p* = 0.0007).

Additionally, we did multiple logistic regression analyses to identify factors associated with the treatment response after 12 months of UDCA therapy (Table 5). Lower pretreatment total bilirubin (OR 0.2777, 95%CI 0.1288–0.5228), ALT (OR 0.5968, 95%CI 0.4354–0.7963), AST (OR 0.4161, 95%CI 0.2736–0.6076), ALP (OR 0.4676, 95%CI 0.3487–0.6048), APRI (OR 0.2838, 95%CI 0.1433–0.5141), as well as higher pretreatment albumin (OR 1.2359, 95%CI 1.1257–1.3714) and platelets (OR 1.0056, 95%CI 1.0011–1.0103) were after age and sex adjustment associated with higher probability of treatment response after 12 months of UDCA therapy. 

Among 190 patients, who were evaluated for treatment response after both 6 and 12 months, there were 132 responders after 6 months (69.5%) and 149 responders after 12 months of UDCA therapy (78.4%, McNemar’s test *p* = 0.004). Seven patients lost initial response (3.7%) and, on the other hand, 24 patients (12.6%) achieved response only after 12 months of UDCA therapy (late responders). There was significant difference in baseline ALP between early and late responders (*p* < 0.00001). Furthermore, there was no difference in ΔAPRI (APRI_PRE-TREATMENT_-APRI_12 MONTHS_, *p* = 0.53) between “12-month responders” and “12-month non-responders”. Treatment response after 6 months of UDCA therapy was significantly associated with treatment response after 12 months of UDCA therapy (OR = 25.2976, 95% CI 10.5881–68.4917, *p* < 0.00001).

We confirmed that both treatment responses after 6 and 12 months of UDCA therapy decrease the risk of an episode of liver decompensation in PBC patients (OR 12.1156, 95%CI 3.7192–54.4826 and OR 21.6000, 95%CI 6.6319–97.3840, respectively; Table 6).

Finally, there was significant difference in both transplant-free and the overall survival distribution between responders and non-responders after 12 months of UDCA therapy (*p* value of the log-rank tests <0.00001 and =0.0008, respectively).

## 4. Discussion

UDCA is the first-line pharmacotherapy in patients with PBC. The first-line treatment aims to stop the progression of the disease, to prevent both liver decompensation and development of hepatocellular carcinoma, and to improve health-related quality of life. Several different treatment response definitions have been proposed so far, and none of them is currently considered to be the gold standard [10,11,12,13,14,15,16]. We used modified Toronto criterion defining the response as ALP ≤ 1.67 × ULN and TB ≤ 2 × ULN—because the Toronto criterion is currently used for indicating the second-line treatment—and evaluated patients’ response after 6 and 12 months of UDCA treatment [12,14]. Patients with Paris-I response (ALP < 3 × ULN, AST < 2 × ULN a TB ≤ 1 mg/dL) have significantly higher 10-year transplantation-free survival [11]. Patients with the Barcelona response (ALP decrease > 40% of baseline values or normal levels after one year of treatment with UDCA) have similar survival to that of patients without PBC. By contrast, PBC patients not responding in such way, have shorter transplantation-free survival [15]. Histological features remained stable in most of the patients who achieved Barcelona response, and it surprisingly improved in some of them. On the other hand, and particularly interesting, histological progression was observed in some patients despite responding to the treatment. No histological improvement was observed among non-responders, on the contrary, histological progression dominated in this group of patients [14]. 

Several different factors may affect patients’ response to UDCA treatment: genetics and epigenetics, gender, age at treatment initiation, AMA status, disease course (symptomatic or asymptomatic), inflammation markers, stage of liver fibrosis, cholestasis, and synthetic function of the liver. We did not seek to investigate the genetics and epigenetics of PBC in this study. The response rate to UDCA is generally lower in the male population of PBC patients. Furthermore, the response rate among patients older than 70 years is about 90%, compared to roughly 50% in patients younger than 30 years [17]. We did not observe a significant difference in response rates between male and female patients nor between patients younger and older than 40 years. That could be due to small number of male patients and patients younger than 40 years (both groups accounted only for 6% of all patients), which could have affected statistical analyses. Furthermore, we need to highlight that in Central and Eastern Europe, male patients account for a smaller proportion of patients when compared to reports from either Scandinavia or North America [2]. The response rate in AMA positive patients is similar to that in AMA negative patients, however, AMA negative patients have worse health-related quality of life [2,6,18]. We have identified 33 AMA negative patients only (13.25%), and, thus, we did not compare response rates between these two groups. During UDCA treatment, symptomatic patients have increased levels of both aminotransferases and alkaline phosphatase, decreased response rate, and increased occurrence of liver cirrhosis, and its complications when compared to asymptomatic patients. For that reason, patients must be diagnosed before they develop symptoms [19]. Furthermore, treatment delay is also associated with decreased response rates to UDCA treatment [20].

After age and sex adjustment, increased baseline levels of AST and ALT were associated with lower probabilities of response after 6 and 12 months of UDCA treatment. In general, aminotransferase levels correlate not only with degree of chronic inflammation but also with degree of liver fibrosis. In PBC, however, these correlations have not been confirmed thus far. There are other factors, which may be of importance in predicting the treatment response. A high neutrophils–to-lymphocytes ratio is an independent predictor of reduced transplant-free survival [21]. PBC is a chronic cholestatic liver disease, and, therefore, it is understandable that in this and all other similar reports, patients with higher levels of ALP had worse response to UDCA treatment after both 6 and 12 months [11,20]. Low ALT/ALP was associated with good response after age and sex adjustment after six months of UDCA therapy. 

Histological features and particularly degree of liver fibrosis correlate with the response to UDCA treatment [22]. In patients with PBC, liver fibrosis can be assessed using different approaches like histology, elastography, or using biochemical and hematological surrogates as well [11,23,24]. Strong correlations were observed between histological assessment and elastography approach, specifically in higher degrees of liver fibrosis [23]. The severity of liver fibrosis—assessed by either histological examination or using laboratory surrogates—predicts treatment response to UDCA [11,24]. In our study, APRI predicted treatment response after age and sex adjustment, on the other hand, AST/ALT ratio did not. That is only partially consistent with another multicentric study where both APRI and AST to ALT ratio predicted response to UDCA treatment [24]. Both transient and magnetic elastography predict liver decompensation in patients on UDCA treatment [25]. Furthermore, both interface hepatitis and ductopenia decrease the chances for treatment response [11]. 

In addition to that, markers of synthetic and detoxification functions of the liver may help predict response to UDCA treatment as well. We confirmed that, after age and sex adjustment, both higher albumin and lower total bilirubin are associated with the response after 6 and 12 months of UDCA treatment. Furthermore, in multiple analyses, lower levels of total cholesterol were associated with the response after six months of UDCA treatment only. Similar results were reported for both total bilirubin and albumin and albumin only by French and Israeli study, respectively [11,26]. Lower levels of Vitamin D could serve as another predictor for treatment response for patients on UDCA [27]. 

There are few more complex possibilities for evaluating treatment efficacy. Currently, there are two kinds of these more complex models. The first depends on the pretreatment data only. In a multicentric study, Carbone et al. developed a model using age and values of aminotransferase, ALP, total bilirubin at diagnosis, treatment time lag (from diagnosis to treatment), and the corresponding ΔALP. The authors report AUROC of 0.87 (95% CI 0.86–0.89) and 0.83 (95% CI 0.79–0.87) on internal and external validation, respectively. GLOBE score and UK-PBC risk score both evaluate biochemical and hematological values after one year of treatment in addition to data from the baseline. The advantage and the disadvantage of these kind of models is that they work with data after one year of treatment. They consider the effect of the treatment itself; thus, they may not be used earlier than after one year of treatment [28,29]. 

Furthermore, we observed that treatment failure after six months of UDCA therapy is associated with 12 times higher chances of liver decompensation (ascites, hepatic encephalopathy, or variceal bleeding) and that treatment failure after 12 months of UDCA therapy is associated with 22 times higher chances of liver decompensation. This finding corresponds with other reports [12].

The vast majority of PBC patients achieve treatment response after six months of UDCA therapy, and virtually all these responders retain response even after 12 months of treatment. Only a small part of PBC patients are “late responders” who do not respond until after 12 months of UDCA therapy. That may be important in the management of PBC patients on UDCA. Further analyses of higher number of six-months PBC non-responders is required for more precise identification of patients, who are at risk of failing to respond even after 12 months of UDCA therapy because specially they are at higher risk for histological progression [14]. Thus, to prevent that from happening, it would be reasonable to add the second-line treatment as soon as after six months of monotherapy (addition of either fibrates or OCA). However, ultimately the best way to treat PBC patients would be to discriminate them at the time of diagnosis using pretreatment characteristics only and treating potential non-responders using UDCA + OCA/bezafibrate from the beginning.

On the whole, this is the first report from countries of Central and Eastern Europe evaluating response to UDCA treatment. We recognize few limitations to this report. First, the retrospective nature of the study, and second, we used a relatively small cohort of patients despite the multicentric design. Furthermore, we did not evaluate histological features in the vast majority of patients, and we dealt with small numbers of both male and young patients, although, this was due to a low proportion of both male and young patients in general. On the other hand, the advantage of our study is that we evaluated response after both 6 and 12 months of UDCA treatment. Non-responders have a significantly increased risk for liver decompensation and, thus, either OCA or fibrates should be added to UDCA monotherapy as soon as possible. 

## 5. Conclusions

We confirmed that in PBC patients, biochemical features (aminotransferases, ALP, total bilirubin, albumin, and ALT/ALP) predict response after 6 and 12 months of UDCA treatment. The vast majority of responders after six months retain response after 12 months of treatment. 

## Figures and Tables

**Table 1:** Patients’ characteristics.

Patients Included		*n* (%)	249 (100%)
Patients evaluated for the treatment response to UDCA	after 6 months after 12 months after both 6 and 12 months	*n* (%)	207 (83.1%) 216 (86.8%) 190 (76.3%)
Gender	Men women	*n* (%)	15 (6%) 234 (94%)
Women ≤ 40 years		*n* (%)	15 (6.4% of female patients, 62% of all patients)
Age	Years	median (IQR)	56.00 (13)
Significant liver fibrosis (APRI > 0.7)		*n* (%)	79 (31.7%)
Follow up	Years	median (IQR)	5 (6)
Liver cirrhosis decompensations		*n* (%)	20 (8%)
- Liver transplantations - Liver-related deaths			- 5 (25% of decompensated patients) - 10 (50% of decompensated patients)
UDCA dosage	mg per day	median (IQR)	1000 (500)

mg = milligrams, *n* = number, SD = standard deviation, UDCA = ursodeoxycholic acid.

**Table 2:** Pretreatment differences in clinical and demographic characteristics between responders and non-responders (response evaluated after 6 months of UDCA treatment).

207 Patients			Non-Responders 66 (31.9%)	Responders 141 (68.1%)	*P*
Age at diagnosis	years	median (IQR)	56.00 (14.75)	54.00 (14.00)	0.77
Gender	Female patients male patients	number (%)	63 (30.4) 3 (1.5)	130 (62.8) 11 (5.3)	0.57
**Total bilirubin**	**×ULN**	**median (IQR)**	**0.59 (0.89)**	**0.52 (0.21)**	**0.03**
**Albumin**	**g/L**	**median (IQR)**	**42.00 (5.32)**	**43.00 (3.16)**	**0.005**
Platelets	×10^9^/L	median (IQR)	231.50 (108.50)	245.00 (97.00)	0.13
Glycemia	mmol/l	median (IQR)	5.41 (1.05)	5.39 (0.94)	0.20
**ALT**	**×ULN**	**median (IQR)**	**1.91 (1.94)**	**1.20 (0.82)**	**0.0001**
**AST**	**×ULN**	**median (IQR)**	**1.68 (1.91)**	**1.10 (0.63)**	**0.00002**
**ALP**	**×ULN**	**median (IQR)**	**2.84 (3.10)**	**1.43 (0.75)**	**<0.00001**
AST/ALT		median (IQR)	0.99 (0.43)	0.96 (0.42)	0.810
**ALT/ALP**		**median (IQR)**	**0.46 (0.46)**	**0.72 (0.52)**	**0.00004**
**TC**	**mmol/L**	**median (IQR)**	**6.18 (2.20)**	**5.96 (1.37)**	**0.03**
**APRI**		**median (IQR)**	**0.74 (0.82)**	**0.43 (0.33)**	**0.00003**

ALP = alkaline phosphatase, ALT = alanine aminotransferase, APRI = AST(ULN)/platelets(×10^9^), AST = aspartate aminotransferase, g/l = grams per liter, IQR = interquartile range, mmol/l = millimoles per liter, SD = standard deviation, TC = total cholesterol, UDCA = ursodeoxycholic acid, ULN = upper limit of normal. Bold highlights statistically significant findings.

**Table 3:** Age and sex adjusted associations of pretreatment clinical characteristics of PBC patients with the treatment response after 6 months of UDCA therapy (multiple logistic regression model).

	OR	95% CI	*p* Value
**Total bilirubin (×ULN)**	**0.3388**	**0.1671–0.6077**	**0.001**
**Albumin (g/L)**	**1.1612**	**1.0706–1.2688**	**0.0005**
Platelets (×10^9^/L)	1.0036	0.9993–1.0080	0.10
Glycemia (mmol/L)	0.8717	0.7298–1.0187	0.10
**ALT (×ULN)**	**0.5306**	**0.3830–0.7080**	**0.00005**
**AST (×ULN)**	**0.4065**	**0.2690–0.5834**	**<0.00001**
**ALP (×ULN)**	**0.3440**	**0.2356–0.4723**	**<0.00001**
AST/ALT	0.8810	0.3916–2.0264	0.76
**ALT/ALP**	**2.4596**	**1.2095–5.5472**	**0.02**
**TC (mmol/L)**	**0.7730**	**0.6242–0.9271**	**0.01**
**APRI**	**0.3375**	**0.1833–0.5774**	**0.0002**

ALP = alkaline phosphatase, ALT = alanine aminotransferase, APRI = AST(ULN)/platelets(×10^9^), AST = aspartate aminotransferase, g/L = grams per liter, mmol/L = millimoles per liter, OR = odds ratio, TC = total cholesterol, UDCA = ursodeoxycholic acid, ULN = upper limit of normal, 95%CI = 95% confidence interval. Bold highlights statistically significant findings.

**Table 4:** Pretreatment differences in clinical and demographic characteristics between responders and non-responders (response evaluated after 12 months of UDCA treatment).

216 Patients			Non-Responders 50 (23.2%)	Responders 166 (76.9%)	*p* Value
Age at diagnosis	years	median (IQR)	55.50 (11)	56.00 (12)	0.30
Gender	female patients male patients	number (%)	49 (22.7) 1 (0.5)	153 (70.8) 13 (6)	0.25
Total bilirubin	×ULN	median (IQR)	0.65 (0.91)	0.52 (0.23)	0.07
**Albumin**	**g/L**	**median (IQR)**	**41.99 (6.35)**	**43.00 (3.04)**	**0.001**
Platelets	×10^9^/L	median (IQR)	236.00 (124.25)	245.00 (93.50)	0.06
Glycemia	mmol/L	median (IQR)	5.24 (0.78)	5.40 (1.10)	0.80
**ALT**	**×ULN**	**median (IQR)**	**1.72 (1.60)**	**1.17 (0.90)**	**0.001**
**AST**	**×ULN**	**median (IQR)**	**1.72 (1.69)**	**1.06 (0.63)**	**0.0001**
**ALP**	**×ULN**	**median (IQR)**	**2.93 (2.73)**	**1.51 (1.01)**	**<0.00001**
AST/ALT		median (IQR)	0.97 (0.55)	0.96 (0.40)	0.58
**ALT/ALP**		**median (IQR)**	**0.49 (0.37)**	**0.70 (0.49)**	**0.0007**
TC	mmol/L	median (IQR)	6.07 (2.08)	5.98 (1.29)	0.56
**APRI**		**median (IQR)**	**0.72 (0.97)**	**0.45 (0.36)**	**0.0001**

ALP = alkaline phosphatase, ALT = alanine aminotransferase, APRI = AST(ULN)/platelets(×10^9^), AST = aspartate aminotransferase, g/L = grams per liter, mmol/L = millimoles per liter, OR = odds ratio, TC = total cholesterol, UDCA = ursodeoxycholic acid, ULN = upper limit of normal, 95%CI = 95% confidence interval. Bold highlights statistically significant findings.

**Table 5:** Age and sex adjusted associations of pretreatment clinical characteristics of PBC patients with the treatment response after 12 months of UDCA therapy (multiple logistic regression model).

	OR	95% CI	*p* Value
**Total bilirubin (×ULN)**	**0.2777**	**0.1288–0.5228**	**0.0004**
**Albumin (g/L)**	**1.2359**	**1.1257–1.3714**	**0.00002**
**Platelets (×10^9^/L)**	**1.0056**	**1.0011–1.0103**	**0.02**
Glycemia (mmol/L)	0.9494	0.7970–1.1575	0.57
**ALT (×ULN)**	**0.5968**	**0.4354–0.7963**	**0.0007**
**AST (×ULN)**	**0.4161**	**0.2736–0.6076**	**0.00002**
**ALP (×ULN)**	**0.4676**	**0.3487–0.6048**	**<0.00001**
AST/ALT	0.6137	0.2544–1.5115	0.28
ALT/ALP	2.1896	1.0093–5.4160	0.07
TC (mmol/L)	0.9604	0.7707–1.2082	0.72
**APRI**	**0.2838**	**0.1433–0.5141**	**0.0001**

ALP = alkaline phosphatase, ALT = alanine aminotransferase, APRI = AST(ULN)/platelets(×10^9^), AST = aspartate aminotransferase, g/L = grams per liter, mmol/L = millimoles per liter, OR = odds ratio, TC = total cholesterol, UDCA = ursodeoxycholic acid, ULN = upper limit of normal, 95%CI = 95% confidence interval. Bold highlights statistically significant findings.

**Table 6:** Association of response to UDCA treatment in PBC patients and the history of liver decompensation (simple logistic regression).

Treatment Response	OR	95% CI	*p* Value
after 6 months of UDCA therapy	12.1156	3.7192–54.4826	0.0002
after 12 months of UDCA therapy	21.6000	6.6319–97.3840	<0.00001

OR = odds ratio, UDCA = ursodeoxycholic acid, 95%CI = 95% confidence interval.

## Data Availability

The data used to support the findings of this study are available from the corresponding author upon request.
